# The Role of Cancer-Associated Fibroblast as a Dynamic Player in Mediating Cancer Stemness in the Tumor Microenvironment

**DOI:** 10.3389/fcell.2021.727640

**Published:** 2021-10-25

**Authors:** Jia Jian Loh, Stephanie Ma

**Affiliations:** ^1^School of Biomedical Sciences, Li Ka Shing Faculty of Medicine, The University of Hong Kong, Hong Kong, Hong Kong; ^2^State Key Laboratory of Liver Research, The University of Hong Kong, Pokfulam, Hong Kong, SAR China

**Keywords:** inflammation, targeted therapy, tumor microenvironment, cancer associated fibroblast, cancer stem cell, cancer stemness

## Abstract

The enrichment of cancer-associated fibroblast (CAFs) in a tumor microenvironment (TME) cultivates a pro-tumorigenic niche *via* aberrant paracrine signaling and matrix remodeling. A favorable niche is critical to the maintenance of cancer stem cells (CSCs), a population of cells that are characterized by their enhanced ability to self-renew, metastasis, and develop therapy resistance. Mounting evidence illustrates the interplay between CAF and cancer cells expedites malignant progression. Therefore, targeting the key cellular components and factors in the niche may promote a more efficacious treatment. In this study, we discuss how CAF orchestrates a niche that enhances CSC features and the potential therapeutic implication.

## Introduction

Tumors are illustrated as “wounds that do not heal” due to the enrichment of fibroblasts and immune cells at the tumor site, which highly mimics that of an inflammatory response of a non-neoplastic tissue (Dvorak, 1986). Indeed, cancer-associated fibroblast (CAF) constitutes the bulk of the stromal component in solid tumors. Cancer cells hijack the normal physiological function of activated fibroblast functions during wound recovery to fuel malignant development. Given cancer stemness accelerates disease progression and impinges therapy, understanding how CAFs cultivate a niche that bestows cancer stem cell (CSC) features in cancer cells may facilitate novel therapeutic intervention.

## Cancer-Associated Fibroblast as a Dynamic Player in Promoting Cancer Stemness

**FIGURE 1:**
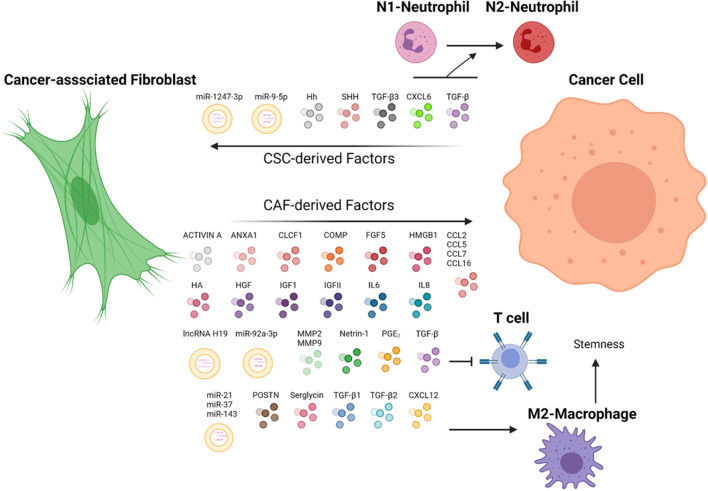
CAF and cancer cells in a dynamic TME. CAF secretes chemokines that can potentiate stemness and metastasis properties of cancer cells *via* distinct pathways. In turn, cancer cells have demonstrated the capacity to direct CAF to fuel their malignant properties. Immune cells, such as T cells, macrophages, and neutrophils, are orchestrated by CAF-cancer cell interaction to promote tumor progression. ANXA1, Annexin A1; CLCF1, cardiotrophin-like cytokine factor 1; COMP, cartilage oligomeric matrix protein; CXCL6, C-X-C motif chemokine ligand 6; CXCL12, C-X-C motif chemokine ligand 12; CCL2, CC-chemokine ligand 2; CCL5, CC-chemokine ligand 5; CCL7, CC-chemokine ligand 7; CCL16, CC-chemokine ligand 16; FGF5, fibroblast growth factor 5; HA, hyaluronan; Hh, hedgehog ligand; HMGB1, high-mobility group box protein 1; HGF, hepatocyte growth factor; IGFI, insulin-like growth factor 1; IGFII, insulin-like growth factor 2; IL6, interleukin 6; IL8, interleukin 8; MMP2; matrix metalloproteinase-2; MMP9, matrix metalloproteinase-9; POSTN, periostin; PGE_2_, prostaglandin E_2_; SHH, sonic hedgehog ligand; TGF-β, transforming growth factor-β; TGF-β1, transforming growth factor-β 1; TGF-β2, transforming growth factor-β 2; TGF-β3, transforming growth factor-β 3; WNT3a, Wnt Family Member 3A. The figure is created with BioRender.com.

Cancer-associated fibroblast, also known as tumor-associated fibroblast, is generally regarded as fibroblasts that exist within or enveloping the tumor (Öhlund et al., 2014). The presence of CAFs throughout tumor development, from the incipient stage to advanced stages, alludes to CAF participation in tumor initiation and progression (Erez et al., 2010). Compared with non-tumor fibroblast (NF) or peri-tumor fibroblast, CAFs are located more proximal to the neoplastic region and exhibit a greater activation marker, namely actin smooth muscle (αSMA) and fibroblast activation protein (FAP) (Qin et al., 2016, 2019). However, compiling studies indicate CAF displays vast heterogeneity within a tumor with distinct functions, underscoring the importance of identifying the function of how each subtype contributes to malignancy (Öhlund et al., 2017; Costa et al., 2018; Lambrechts et al., 2018). A myriad of elements may affect CAF subtypes, for instance, proximity to the tumor. CAF subsets were distinguished based on their expression of α*SMA* and *IL6* with high-α*SMA* localized nearer to the tumor and high-*IL6* further, indicating juxtacrine and paracrine interaction between cancer cells and fibroblast may stimulate the CAF to differentiate to subtypes with distinct functions (Öhlund et al., 2017). Aside from molecular markers, the secretome of CAF and NF can be discerned as the former secretes a greater abundance of ligands that promote stemness properties of HCC (Jiang et al., 2017; Álvarez-Teijeiro et al., 2018). CAF plays an extensive role in shaping the extracellular matrix (ECM) of the tissues, allowing it to exert its oncogenic influence by modulating the physical properties of the TME. In addition to promoting cancer progression, fibroblasts are shown to be educated by neoplastic cells to their benefits, depicting the dynamic cellular network in the TME (Figure 1).

Cancer stem cells are generally deemed as a rare population of cancer cells that share similar features to normal stem cells, including the ability to self-renew and differentiate into lineages that comprise the tumor bulk, thus regarded as tumorigenic, as opposed to non-CSC cancer cells (Reya et al., 2001; Clarke et al., 2006). Common CSC markers include surface markers, such as CD24, CD44, CD90, CD133, EpCAM, LGR5, and aldehyde dehydrogenase (ALDH) ALDH activity (summarized in Walcher et al., 2020). Moreover, expressions of stemness genes, such as *OCT4*, *SOX2*, and *NANOG*, are often deployed to measure cancer stemness. Functional surrogate to assess cancer stemness properties includes *in vitro* sphere formation and *in vivo* limiting dilution in which cancer cells are injected at low doses into animal models. Cancer cells with greater stemness are generally considered to have augmented metastasis ability due to their enhanced ability to reconstitute a tumor at the secondary site, and the acquisition of EMT is coupled with elevated stem cell properties, including stem cell markers and tumorigenicity, signifying EMT mimics features of CSC (Mani et al., 2008; Baccelli and Trumpp, 2012; Oskarsson et al., 2014). Emerging evidence illustrates that stemness properties of cancer cells are dependent on their niche (Plaks et al., 2015; Batlle and Clevers, 2017). Factors derived from niche may promote plasticity of non-CSC cancer cells into CSC, whereas depletion of factors may reduce the CSC population, thereby implicating niche factors as potential therapeutic targets (Batlle and Clevers, 2017). Given the CAF fosters the niche by enriching pro-tumor factors and remodeling the matrix of the TME, understanding how a niche facilitates cancer initiation and progression will allow us to contrive therapy targeting the key cellular components and factors sustaining cancer stemness.

### Cancer Cells Educate Cancer-Associated Fibroblast to Adopt a Pro-tumor Phenotype

Accumulating research illuminates the cancer cells instigate pro-tumor CAF to foster a pro-favorable niche. Transforming growth factor-β3 (TGF-β3) derived from HNSCC cells can activate CAF to secrete POSTN, leading to greater metastasis ability of the neoplastic cells (Qin et al., 2016). Prostate cancer cells can recruit marrow-derived mesenchymal stem cells (MSCs) and activate them into CAF by TGF-β1 (Barcellos-de-Souza et al., 2016). Of significance, MSC-derived CAF can recruit and induce monocytes into M2 macrophages, illustrating the CAF capacity to govern the TME. Cardiotrophin-like cytokine factor (CLCF1) derived from CAF stimulates the production of C-X-C motif ligand 6 (CXCL6) and TGF-β, which not only escalate stemness properties of cancer cells but also stimulate CLCF1 expression in the CAF, thereby fostering a positive feedback loop that expedites malignancy (Song et al., 2021). Furthermore, CXCL6 and TGF-β can enhance infiltration and development of pro-tumor N2-neutrophils (Song et al., 2021), corroborating CAF capacity to foster an immunosuppressive TME. Sonic hedgehog (SHH) ligand derived from CD24^+^CD49f^hi^breast CSCs constructs a pro-tumorigenic TME by activating CAF (Valenti et al., 2017). Cancer cells-derived hedgehog (Hh) instigates the production of pro-tumor paracrine factors and ECM constituents in CAF (Cazet et al., 2018). Autocrine and colorectal cancer cells-derived paracrine signaling of IL34 facilitate the conversion of normal fibroblast into CAF that adopts a pro-tumor secretome that includes stemness-promoting factors, such as Netrin-1 and FGF2 protein (Giulianelli et al., 2019; Sung et al., 2019). The coculture of HNSCC cells and CAF stimulate CAF to generate WNT3A, leading to increased cancer stemness (Le et al., 2019).

Exosomal miRNAs are gaining interest as a mediator between cancer cell-fibroblast crosstalk. Activation of focal adhesion kinase (FAK) signaling by cancer cell-derived miRNA stimulates the production of CAF-derived exosomes that facilitate the spheroid formation and metastasis (Wu et al., 2020). Tumor-derived exosomal miR-1247-3p triggers the activation of fibroblast by fueling NF-κB signaling consequently stimulates the CAF to secrete a greater abundance of IL6 and IL8, instigating the cancer cells to an EMT phenotype with enhanced lung metastases (Fang et al., 2018). Exosomal miR-9-5p elevates IL6 production in CAF, leading to enhanced spheroid-forming ability (Zhang et al., 2020). In summary, targeting the aforementioned factors or exosomes may disrupt the ability of cancer cells to assemble a pro-tumor niche.

### Cancer Cell-Educated Cancer-Associated Fibroblast Cultivates a Supportive Secondary Site for Colonization

The TGF-β family is shown to play pivotal roles in allowing cancer cells to colonize secondary sites. TGF-β3 rooted from cancer cells fortifies a favorable niche to colonize a foreign site by stimulating fibroblast-derived POSTN, which is crucial for initiating breast cancer cell colonization at the lung by maintaining CSC properties *via* the Wnt signaling (Malanchi et al., 2011). In accordance, HNSCC cancer cells secrete TGF-β3 that enhances POSTN secretion from CAF, resulting in the augmented migratory phenotype of cancer cells (Qin et al., 2016). Upon stimulation by TGF-β, CAF confers colorectal cancer cells-enhanced tumor-initiating capacity and reduced tumor latency (Calon et al., 2015). Tumor organoids with elevated TGF-β profoundly enhanced ability to form liver metastases by orchestrating a pro-tumor stroma niche (Calon et al., 2015). Colorectal cancer (CRC) fueled by driver mutations in the *Lgr5* + stem cells is accompanied by enrichment of CAF-derived TGF-β that suppresses the tumor-killing T cell populations, thus leading to greater metastases to the liver (Tauriello et al., 2018), highlighting the interplay between CAF, immune cells, and cancer cells. Conversely, the TGF-β-signaling inhibitor significantly dampened the tumor volume and liver metastases of the colon.

### Paracrine Signaling of Cancer-Associated Fibroblast Enhances Cancer Stem Cell Features

In a tumor, fibroblast-secreted factors and cytokines, which serve for tissue recovery, are seized by cancer cells. Compiling studies have depicted the critical roles of CAF-derived factors in maintaining the CSC features of cancer cells by promoting stemness pathways (Table 1). HGF is reported to enhance cancer stemness by potentiating the frequency of CD44^+^, CD47^+^, and CD90^+^ HCC CSC and spheroid formation, stemness and EMT gene expressions (Lau et al., 2016; Ding et al., 2018; Yan et al., 2018). Mechanistically, HGF exerts its oncogenic influence *via* distinct mechanisms in a tissue-dependent manner. HGF augments ERK/FRA1/HEY1, STAT3/TWIST1, and YAP/HIF-1α in HCC, gastric cancer, and pancreatic cancer, respectively (Lau et al., 2016; Ding et al., 2018; Yan et al., 2018). CAF-derived IL6 is demonstrated to fuel stemness as evident in increased spheroid formation, stemness genes markers *via* STAT3 signaling (Xiong et al., 2018; Zhang et al., 2020). CAF paracrine signaling regulates the AKT pathway *via* insulin-like growth factor 2 (IGFII), thereby promoting *NANOG* expression, enhanced tumorigenicity *in vivo*, and ALDH activity (Chen et al., 2014). The AKT signaling is also mediated by cartilage oligomeric matrix protein (COMP) to fuel EMT and spheroid formation (Li et al., 2018). CC-chemokine ligand 2, 5, 7, and 16 (CCL2, CCL5, CCL7, and CXCL16) potentiate EMT *via* the Hh and TGF-β signaling (Liu et al., 2016). Serglycin (SRGN), a proteoglycan secreted from cancer cells and CAFs, renders chemoresistance and EMT features *via* a CD44-dependent NF-κB activation (Guo et al., 2017). The binding of CAF-derived periostin (POSTN) to protein tyrosine kinase 7 (PTK7) drives the β-catenin signaling, leading to increased expression of CSC markers and stemness genes (Yu et al., 2018). Annexin A1 (AnxA1) secreted from CAF potentiates stemness and EMT genes expressions, and spheroid formation (Geary et al., 2014). Prostaglandin E_2_ (PGE_2_) produced from fibroblasts drives the expansion of tumor-initiating cells by inducing YAP signaling (Roulis et al., 2020). The cellular crosstalk within the TME was displayed as CAFs recruit tumor-associated macrophages (TAMs) *via* CXCL12 secretion and induces the M2-phenotype TAMs, subsequently promotes stemness and EMT signature genes (Li et al., 2019). Alteration in specific genes may dictate the impact of CAF on cancer cells. Androgen receptor (AR)-depleted CAF enhances the production of IFN-γ and M-CSF, leading to increased cancer stemness (Liao et al., 2017). Notch1-depleted CAFs augment CD271^+^ melanoma CSC frequency (Du et al., 2019) and metastasis to the lung (Shao et al., 2016). Together, blocking CAF-derived factors or a downstream signaling cascade may be a therapeutic revenue to diminish cancer stemness.

**TABLE 1:** Cancer-associated fibroblast (CAF)-derived factors on cancer stemness.

Factors	Disease	Mechanism	References
ACTIVIN A and IGF1	Breast cancer	CSC secretes SHH to cultivate pro-tumor CAF which then generates ACTIVIN A and IGF1 to promote stemness*	Valenti et al., 2017
ANXA1	Prostate cancer	Induces EMT and stemness markers	Geary et al., 2014
CCL2, CCL5, CCL7, and CXCL16	Hepatocellular carcinoma	Promotes Hh and TGF-β signaling in HCC cells	Liu et al., 2016
CLCF1	Hepatocellular carcinoma	Promotes AKT/ERK/STAT3 signaling. STAT3 target genes, CXCL6, and TGF-β induce CAF’s CLCF1 secretion, thus forming CAF-cancer cell positive feedback*	Song et al., 2021
COMP	Hepatocellular carcinoma	Promotes ERK and AKT signaling in HCC cells	Li et al., 2018; Sun et al., 2019
CXCL12	Oral squamous cell carcinoma	Induces M2-phenotype of TAMs that promote EMT and stemness in cancer	Li et al., 2019
FGF5	Triple-negative breast cancer	Cancer cell-derived HH induces FGF5 production from CAF; FGF5 elevates stemness genes expression in cancer cells*	Cazet et al., 2018
HGF	Hepatocellular carcinoma	Promotes c-MET/ERK/FRA1/HEY1 axis in HCC cells*	Lau et al., 2016
	Gastric cancer	Promotes c-Met/STAT3/twist1 signaling and EMT signaling	Ding et al., 2018
	Pancreatic Cancer	Promotes c-MET/YAP/HIF-1α signaling	Yan et al., 2018
	Intrahepatic cholangiocarcinoma	Promotes AKT/ERK signaling	Affo et al., 2021
HMGB1	Luminal breast cancer	Promotes stemness markers and ALDH1+ population upon binding to TLR4	Zhao et al., 2017
IGFII	Lung cancer	Promotes IGII/IGF1R/AKT/NANOG signaling	Chen et al., 2014
IL6	Hepatocellular carcinoma	Promotes STAT3/NOTCH1/NICD/HES1 signaling	Xiong et al., 2018
	Intrahepatic cholangiocarcinoma	Promotes STAT3/EZH2 cascade. Neoplastic cell-secreted exosomal miR-9-5p upregulates IL6 production*	Zhang et al., 2020
IL6 and IL8	Head and Neck cancer	Promote chemoresistance	New et al., 2017
	Hepatocellular carcinoma	HCC cell-derived mi-1247-3p stimulate CAF to produce IL6 and IL8, which promotes stemness and EMT*	Fang et al., 2018
	Breast cancer	CD10^+^GRP77^+^ CAF-derived IL6 and IL8 stemness	Su et al., 2018
Netrin-1	Non-small cell lung cancer and colon cancer	Promote stemness marker and ALDH1A expression	Sung et al., 2019
Netrin-1 and FGF2	Colorectal cancer	IL34-acitvated CAF increases Netrin-1 and FGF2 expression	Franze et al., 2020
MMP2 and MMP9	Prostate carcinoma	Cancer cell-derived IL6 activate CAF to produce MMP2 and MMP9, in turn, promote EMT and stemness*	Giannoni et al., 2010
PGE_2_	Intestinal cancer	Promotes pro-oncogenic PTGER4/YAP	Roulis et al., 2020
POSTN	Breast cancer	Activates Wnt signaling	Malanchi et al., 2011
	Head and neck cancer	TGF-β3 activated CAF to secrete POSTN	Qin et al., 2016
	Human head and neck squamous cell carcinoma	Promotes PTK7/GSKβ/β-catenin signaling	Yu et al., 2018
TGF-β	Colorectal cancer	CSC modulates CAF-derived TGF-β to suppress the tumor-killing T cell populations*	Tauriello et al., 2018
TGF-β1	Pancreatic Cancer	Promotes the upregulation of ATF via the SMAD2/3 pathway	Wei et al., 2021
TGF-β2	Colorectal cancer	Upregulates GLI2 expression	Tang et al., 2018
SRGN	Non-small cell lung cancer	Promote stemness gene markers in CD44-dependent manner	Guo et al., 2017
WNT3A	Human head and neck squamous cell carcinoma	Neoplastic cells activate WNT3A production in CAF*	Le et al., 2019
Exosomal miR-21, miR-143, and miR-378	Breast cancer	Enhances stemness and EMT	Donnarumma et al., 2017
Exosomal lncRNA H19	Colorectal cancer	Sponges miR-141 that target β-catenin	Ren et al., 2018
Exosomal miR-92a-3p	Colorectal cancer	Targets FBXW7 to enhance β-catenin activity and MOAP1 to enhance chemoresistance properties	Hu et al., 2019
Exosomal miR-196a	Head and Neck cancer	Targets CDKN1B and ING5, to promote cisplatin resistance	Qin et al., 2019
Exosomal miR-423-5p	Prostate cancer	Promotes chemoresistance by targeting GREM2, leading to TGF-β pathway activity	Shan et al., 2020

*Denotes a cancer cell-to-CAF dialog.

### Cancer-Associated Fibroblast Mediates Cancer Stemness via Extracellular Matrix Remodeling

A fundamental function of activated fibroblast is its participation in ECM homeostasis by synthesizing ECM constituents, including collagen and fibronectin, and producing ECM-degrading proteases to aid communication and trafficking of inflammatory cells (Kalluri, 2016). While the impact of matrix stiffness remains controversial as studies indicate both softer matrix (Tan et al., 2014; Ng et al., 2021) and stiffer matrix (Pang et al., 2016; Tao et al., 2021) can instigate cancer stemness, the role of CAF in mediating the stiffness of the ECM illustrates its ability to affect stemness. Indeed, collagen deposited by CAF functions as a mechano signal to escalate stem cell markers and spheroid formation ability (Cazet et al., 2018). Additionally, hyaluronan (HA), a major constituent of the ECM that boosts CSC self-renewal and EMT (summarized in Chanmee et al., 2015), is shown to be highly produced by CAF (Affo et al., 2021). Given CAFs are a source of ECM-degrading proteases, namely matrix metalloproteinases (MMPs), they can affect CSC properties by remodeling the ECM. Studies demonstrated IL6 derived from prostate cancer cells elicits pro-tumor FAP^+^CAF (Giannoni et al., 2010). Consequently, these FAP^+^ CAF secretes MMP2 and MMP9 to potentiate EMT and stemness genes. MMP3 that is predominantly produced by fibroblast (Witty et al., 1995) is demonstrated to promote stem cell population by enhancing canonical Wnt signaling, potentially leading to hyperplastic growth of normal tissues (Kessenbrock et al., 2013), depicting fibroblast can contribute to tumor initiation *via* ECM remodeling. Future research can focus on ablating the effect of CAF on the ECM, either by targeting ECM constitutes or MMPs, to attenuate the CAF-mediated oncogenic effect.

### Cancer-Associated Fibroblast Role in Endowing Drug Resistance

The augmented cancer stemness conferred by CAF may endow cancer resistance to conventional therapy. Patients with breast cancer, who are resistant to chemotherapy, show a greater abundance of CAF marked with a cluster of differentiation 10 (CD10) and G protein-coupled receptor 77 (GRP77) cell surface proteins (Su et al., 2018). CD10^+^GRP77^+^ CAFs promote sphere formation, the proportion of ALDH^+^ and CD44^+^CD24^–^ breast CSCs, and chemoresistance properties of cancer cells by secreting IL6 and IL8. A similar phenomenon was observed in HNSCC as CAFs undergoing autophagy secrete greater levels of IL6 and IL8 to confer chemoresistance (New et al., 2017). Increased *NANOG* and *SOX2* stemness gene expression in cancer cells treated with CAF-conditioned media may lead to greater cisplatin resistance (Peltanova et al., 2021). CAF-derived TGF-β1 drives self-renewal and gemcitabine resistance by upregulation of activating transcription factor 4 (ATF4) *via* the SMAD2/3 pathway (Wei et al., 2021). SRGN facilitates cisplatin resistance *via* inducing NANOG (Guo et al., 2017). In line, CAF-rooted TGF-β2 can elicit stemness and chemoresistance by enhancing GLI Family Zinc Finger 2 (GLI2) (Tang et al., 2018). CAF-derived exosomes enhance the stemness properties of CD133^+^ colorectal CSCs, leading to greater chemoresistance to fluorouracil and oxaliplatin (Hu et al., 2015). Additionally, exosomal miR-92a-3p derived from colorectal CAF targets anti-tumor F-box/WD repeat-containing protein 7 (FBXW7) and a modulator of apoptosis 1 (MOAP1), thereby endowing chemoresistance to cancer cells (Hu et al., 2019). In line, exosomal miRNA was demonstrated to confer chemoresistance in prostate cancer (Shan et al., 2020) and head and neck cancer (Qin et al., 2019). CAF-derived exosomal long non-coding RNA (lncRNA) H19 was exhibited to enable stemness expression by sponging miR-141 that targets β-catenin (Ren et al., 2018).

Another mechanism that CAF mediates chemoresistance resides in its ability to remodel the ECM. Studies have demonstrated HA upregulates NANOG/STAT signaling, leading to increased expression of multidrug transporter (MDR1) that contributes to chemoresistance (Bourguignon et al., 2008). In accordance, HA-CD44 interaction mediates stemness signaling that governs miRNA regulation of genes involved in chemoresistance in breast cancer cells (Bourguignon et al., 2009) and HNSCC cells (Bourguignon et al., 2012a, b). HA mediates CD44v3^high^ALDH1^high^HNSCC CSC *via* an epigenetic alteration to promote cisplatin chemoresistance (Bourguignon et al., 2016). The ability of CAF to mediate matrix stiffness *via* MMPs may be an area of interest. Given matrix stiffness regulates stemness and chemoresistance (Ng et al., 2021); how a CAF-mediated ECM fosters a niche that endows chemoresistance warrants further investigations.

### Single-Cell RNA Sequencing Identified Cancer-Associated Fibroblast Subsets With Distinct Functions on Cancer Stemness

State-of-the-art single-cell transcriptomic profiling of intrahepatic cholangiocarcinoma (ICC) revealed six distinct fibroblast populations (Zhang et al., 2020). Vascular CAFs (vCAF), the most abundant population, were found to secrete IL6 to promote self-renewal of cancer cells. Of note, the characterization of the function of other CAF subtypes identified such as matrix CAFs, which expressed high ECM signature genes, would be an interesting area of research. Lineage tracing coupled with single-cell RNA sequencing (scRNA-seq) in sophisticated animal models categorized CAF into myofibroblast (myCAF) and inflammatory and growth factor-enriched CAF (iCAF) populations that modulate tumor cell proliferation *via* distinct mechanisms; the former has a high level of hyaluronan synthase 2 (*Has2*) that can enhance pro-tumor HA; the latter secretes HGF (Affo et al., 2021). scRNA-seq identified prostaglandin-endoperoxide synthase 2 (Ptgs2)-expressing fibroblasts that expand tumor-initiating stem cells *via* YAP signaling, whereas ablation of Ptgs2 diminished the occurrence of the small intestine and colon cancers (Roulis et al., 2020). Capturing cellular diversity at single-cell resolution ignites exciting research questions as follows: how cellular and non-cellular components of the TME govern CAF identity? How each subtype contributes to disease progression? How CAFs differentiate or undifferentiate into different subtypes?

## Potential Therapeutic Strategy and Challenges

### Targeting Multiple Cancer-Associated Fibroblast-Derived Factors to Circumvent Stemness

As aforementioned (Table 1), cancer-promoting bioactive molecules derived from stromal cells may be targeted using specific neutralizing antibodies, particularly in combination with chemotherapy. However, the promising preclinical results have yet to yield optimistic results in clinical settings. A Phase 2 trial (NCT00433446) examining siltuximab (CNTO328), an anti-IL6 antibody, showed siltuximab did not have clinical benefit for patients with advanced prostate cancer (Dorff et al., 2010). Similarly, the addition of siltuximab (CNTO328) to mitoxantrone/prednisone regimen did not yield improved clinical (NCT00385827) outcomes compared to mitoxantrone/prednisone alone in treating advanced prostate cancer (Fizazi et al., 2012). The Phase 2, single-arm, clinical trial (NCT00992186) explored the efficacy of carlumab (CNTO 888), antibody targeting (CCL2), in patients with metastatic castration-resistant prostate cancer (CRPC), who had undergone docetaxel treatment (Pienta et al., 2013). However, none of the patients treated with carlumab showed partial or complete remission. One key possibility is that, while the CCL2 level was depleted upon carlumab administration, the level rebounded rapidly within a week, thus suggesting carlumab cannot suppress CCL2 for the clinically meaningful duration (Pienta et al., 2013). Nonetheless, the addition of siltuximab to bortezomib-melphalan-prednisone (VMP) demonstrated marginal clinical benefits in multiple myeloma by statistically improving partial response rate (San-Miguel et al., 2014). Together, these imply more precise stratification of patients and usage of biomarkers may result in better clinical outcomes. Of note, several clinical trials are exploring the clinical utility of neutralizing antibodies that target HGF (NCT04368507), MET (NCT04077099), and IL6R (NCT03999749). Aside from neutralizing antibodies, a combination of inhibitors-targeting receptors, including Osimertinib (an EGFR inhibitor) and Savolitinib (MET inhibitors), are being tested in clinical trials (NCT03778229). Notably, MP0250, a drug candidate targeting HGF and VEGF, which have met the safety requirement and demonstrated clinical efficacy, is being tested in combination with existing drugs to treat multiple myeloma (NCT03136653). Given the TME is often accompanied by an upsurge of cytokines and chemokines, targeting multiple factors may promote treatment efficacy.

### Depleting Pro-tumor Cancer-Associated Fibroblast

The ability of CAF to endow stemness of cancer cells makes it an intriguing therapy. CAF is shown to be more positively correlated with gene sets associated with poor prognosis compared with epithelial cancer cells, immune cells, and endothelial cells (Calon et al., 2015), substantiating targeting CAF as a potential therapeutic avenue. Studies have demonstrated that α*SMA* expression represents a marker for worse prognosis in colorectal cancer, indicating myofibroblast abundance is crucial to disease prediction (Tsujino et al., 2007). In accordance, studies conducted in tongue cancer and oral cancer depicted similar results (Vered et al., 2010; Marsh et al., 2011).

However, the heterogeneity of CAF necessitates precise identification of more specific markers as tumor-restraining CAF exists. Genetic deletion of α*SMA* in mouse models enhances the progression of pancreatic ductal adenocarcinoma (PDAC) as evidenced by a lower survival rate in α*SMA*-depleted mice (Ozdemir et al., 2014). Interestingly, the myofibroblast-depleted tumor demonstrated enhanced spheroid-forming ability, indicating a greater proportion of CSC. Indeed, identification of CAF subtypes shows they promote or suppress tumor progression in a tissue-dependent manner (Galbo et al., 2021), corroborating the need for further investigation into more CAF-specific markers. Recent work has illuminated the potential therapeutic benefit of targeting tumor-promoting fibroblasts. Administration of GRP77 neutralizing antibody-targeting CAF depletes CAF-secreted IL6 and IL8, thereby abolishing a stem cell-supporting niche and sensitizing breast cancer cells to doxycycline, leading to significant shrinkage of the tumor volume (Su et al., 2018), highlighting the therapeutic potential of targeting precise tumor-promoting CAF.

### Rewire Pro-tumor Cancer-Associated Fibroblast Into Quiescent or Anti-tumor Fibroblast

Reeducating pro-tumor CAF into a quiescent state or even anti-tumor CAF is a tempting strategy. Vitamin D metabolite 1α, 25-dihydroxyvitamin D3 [1,25(OH)2D3] is shown to deplete the oncogenic influence of stromal fibroblast to the cancer cells, while 1,25(OH)2D3-treated stromal fibroblast displayed a gene signature that favors a clinical outcome (Ferrer-Mayorga et al., 2017). VDR activation of the stromal fibroblast using calcipotriol, a vitamin D analog, diminished expression of genes involved in growth factors and cytokines, for instance, the *IL6* and *POSTN*, thereby suppressing a tumor-promoting secretome (Sherman et al., 2014). Additionally, the combination treatment of calcipotriol and gemcitabine markedly prolonged survival in preclinical models. Moreover, high vitamin D receptor (VDR) expression in stromal cells predicts favorable survival (Ferrer-Mayorga et al., 2017). All-trans retinoic acid (ATRA), also known as tretinoin, a vitamin A metabolite, renders pancreatic stellate cells into a quiescent state, which, in turn, secreted a greater level of secreted frizzled-related protein 4 (sFRP4) that negatively modulates Wnt signaling of cancer cells in a paracrine manner (Froeling et al., 2011). Indeed, ATRA has passed the safety in Phase I clinical trial (NCT03307148) and will proceed to Phase II (NCT04241276) as encouraging therapeutic responses were observed (Kocher et al., 2020). Given vitamins are essential for healthy tissue and their toxicity is relatively lower compared to chemotherapy, repurposing vitamin analogs to rewire activated fibroblast into a quiescent state may present a viable therapeutic strategy that can be translated into the clinical settings.

Another strategy to rewire the population of fibroblasts is by reprogramming the fibroblast using growth factors as exemplified in the plasticity of CAF found in PDAC. While IL1 induces fibroblast into having an inflammatory phenotype categorized by an elevated cytokines production, TGF-β antagonizes an IL1-induced phenotype and stimulates the fibroblast to adopt a myofibroblastic phenotype with less tumorigenesis, particularly reduced expression of factors promoting cancer stemness such as *Il6* and *Cxcl12* (Biffi et al., 2019). Together, this rationalizes the option to rewire tumor-promoting into tumor-restraining fibroblast. To account for the ability of the cancer cell to instigate tumor-promoting CAFs, inhibitors may be deployed to circumvent cancer cell-mediated activation of CAF. Breast cancer cells activate CAF *via* the hedgehog signaling to cultivate a stem cell niche by ECM remodeling and FGF5 secretion that promote docetaxel chemoresistance (Cazet et al., 2018). As such, targeting the crosstalk *via* smoothened inhibitors (SMOi), a hedgehog-signaling inhibitor, sensitizes triple-negative breast cancer (TNBC) to docetaxel. Concomitantly, inhibition of hedgehogs abrogates the activated stromal cells, thereby augmenting the efficacy of chemotherapy in treating pancreatic cancer (Olive et al., 2009). However, either genetic depletion or pharmacological inhibition of SHH, a ligand that activates pancreatic CAFs, resulted in less stromal composition but also a more aggressive tumor (Rhim et al., 2014), suggesting treatment should be tailored based on tissue and treatment.

## Future Perspectives

Advances in single-cell RNA sequencing may not only aid the characterization of cell types based on their molecular profiles and, subsequently, functions but also have the potential to be used to identify new biomarkers for patient stratification and tailor-personalized medicine (Dominguez et al., 2020). Harnessing single-cell RNA-sequencing to profile CAF at the molecular level, future studies can address outstanding questions, including the origin and development of specific CAF subtypes, identification of biomarkers corresponding to each subtype, how each CAF subtype interacts with its niche, and amenable therapeutic opportunities to tackle tumor-promoting CAF. A more holistic approach investigating the CAF molecular profile, for instance, epitranscriptomic and epigenetics (Delaunay and Frye, 2019; Song et al., 2020), may unravel novel insights into how CAF shuttles between cell types. Recent studies have unmasked that the altered epigenetics profile between CAF and NF allows the former to generate greater levels of WNT5A that confers malignancy to the neoplastic cells (Maeda et al., 2020). A critical unmet knowledge gap in our understanding of CAF function is if juxtracrine signaling between CAF and cancer cells affects cancer stemness.

Given the TME is a dynamic region with various cell types actively contributing to tumor progression, merely focusing on targeting a single aspect seems unlikely to yield any long-term therapeutic benefit. For example, targeting niche factors alone may not be sufficient to eradicate the tumor due to the inclination of cancer cells to evolve into a niche-independent malignancy as the disease progresses (Fujii et al., 2016). Therefore, comprehensive characterization of cell types and their respective functions in the TME may pave the way for a multimodal approach to improve cancer treatment.

## Author Contributions

JL wrote the review article. SM edited the review article and provided funding support. Both authors contributed to the article and approved the submitted version.

## Conflict of Interest

The authors declare that the research was conducted in the absence of any commercial or financial relationships that could be construed as a potential conflict of interest.

## Publisher’s Note

All claims expressed in this article are solely those of the authors and do not necessarily represent those of their affiliated organizations, or those of the publisher, the editors and the reviewers. Any product that may be evaluated in this article, or claim that may be made by its manufacturer, is not guaranteed or endorsed by the publisher.
